# Exhaled Breath Condensate—A Non-Invasive Approach for Diagnostic Methods in Asthma

**DOI:** 10.3390/jcm10122697

**Published:** 2021-06-18

**Authors:** Joanna Połomska, Kamil Bar, Barbara Sozańska

**Affiliations:** 1st Department and Clinic of Paediatrics, Allergology and Cardiology, Wrocław Medical University, 50-367 Wrocław, Poland; kamilbarmd@gmail.com (K.B.); barbara.sozanska@umed.wroc.pl (B.S.)

**Keywords:** asthma, EBC, exhaled breath condensate, exhaled biomarkers, microbiota

## Abstract

The pathophysiology of asthma has been intensively studied, but its underlying mechanisms such as airway inflammation, control of airway tone, and bronchial reactivity are still not completely explained. There is an urgent need to implement novel, non-invasive diagnostic tools that can help to investigate local airway inflammation and connect the molecular pathways with the broad spectrum of clinical manifestations of asthma. The new biomarkers of different asthma endotypes could be used to confirm diagnosis, predict asthma exacerbations, or evaluate treatment response. In this paper, we briefly describe the characteristics of exhaled breath condensate (EBC) that is considered to be an interesting source of biomarkers of lung disorders. We look at the composition of EBC, some aspects of the collection procedure, the proposed biomarkers for asthma, and its clinical implications. We also indicate the limitations of the method and potential strategies to standardize the procedure of EBC collection and analytical methods.

## 1. Introduction

Recent epidemiological studies revealed that more than 300 million individuals live with asthma worldwide [1], with the prevalence of the disease increasing from the middle of the 20th century in many developing countries [2]. Bronchial asthma imposes an unacceptable burden on society and economy in terms of medication costs, its impact on the quality of life, and high global morbidity, even among young people [3]. Asthma is considered to be the most common chronic respiratory disease in childhood [4]; it often begins in the first years of life and may potentially impair the development of the respiratory system and reduce maximally attained lung function [5]. The current classification of diseases of airways focuses mainly on the description of clinical manifestations, symptoms, triggers, or lung function tests. However, the spectrum of airway disorders with different clinical characteristics is broad, and the underlying pathological mechanisms, including airway inflammation, control of airway tone and reactivity are not completely explained. We presently know that asthma is a complex, heterogeneous disease with variable clinical presentations and prognoses, but different asthma subtypes still need to be identified and adequately described in terms of underlying molecular mechanisms, which would potentially allow for the primary prevention of asthma as well as its targeted treatment.

Nowadays, the concept of biochemical phenotyping and the connection between molecular pathways and clinical manifestations is under intense debate [6]. Researchers have focused on finding different biochemical and metabolic phenotypes and biomarker panels to select patients with mild, moderate, or severe asthma. It would be of great clinical usefulness to find biomarkers that are easy to collect in a non-invasive manner to confirm the diagnosis, assess disease activity, or predict treatment response.

In the pediatric population, the current methods of evaluating the pathology of the lung such as pulmonary function testing (spirometry) and biological sample collection (e.g., induced sputum) are not easy to perform. Moreover, the same clinical picture may be related to different underlying pathophysiological mechanisms [7]. Therefore, it is not easy to study local airway inflammation based only on the results of functional lung tests or induced sputum collection. Specific diagnostic methods such as bronchoalveolar lavage (BAL) or bronchoscopy with bronchial biopsy provide direct information about local airway inflammation, but these procedures are unacceptable for routine practice as they are invasive methods. Some procedures such as peripheral blood/serum collection are minimally invasive and easy to conduct, but serum markers may not always reflect local airway inflammation. Given all these difficulties, there is an urgent need for implementing novel, non-invasive diagnostic methods that can also be applied in children.

In this review, exhaled breath condensate (EBC) is discussed as an easy to collect, non-invasive, and affordable tool to be used in the diagnosis of asthma. We briefly describe the design of EBC, some aspects of the collection procedure, the proposed biomarkers for asthma, and its clinical implications.

## 2. Exhaled Breath Condensate—Definition

Exhaled biomarkers represent a rapidly growing field of research [8] and exhaled breath condensate is a non-invasive tool for the collection of biological samples from the airways. EBC analysis seems to be an increasingly used and promising method in research, as a wide number of inflammatory mediators can be detected in it [9,10,11,12]. The concentrations of these mediators are influenced by lung diseases and modulated by therapeutic interventions. According to the definition by the European Respiratory Society and American Thoracic Society, EBC is a fluid or frozen material obtained by cooling exhaled air through contact with a cold surface of a condenser [13]. EBC is collected during tidal breathing using nose-clip and saliva traps, with defined temperature and collection time, into a condenser made of inert material, without resistor and filter between the subject and the condenser [9]. It contains volatile and non-volatile compounds and is mainly composed of nitrogen, oxygen, carbon dioxide, argon, and water vapor. As material from the bronchial tree may be obtained with the use of other techniques, not only by cooling exhaled breath, the term exhaled breath condensate strictly relates to exhaled samples that are collected by cooling the exhaled breath. In other words, the term exhaled breath condensate cannot be used if the method of obtaining material from the respiratory tract is not based on cooling the exhaled air [9].

## 3. Exhaled Breath Condensate—Collection Devices

Different types of condensing equipment have been designed including commercially available condensers as well as homemade systems (Table 1). Condenser devices differ in some respects such as cooling systems, lining materials of the collecting tube, the possibility of fractionated collection, or even in terms of electric power requirement during sampling [14,15]. The design of the collection equipment prevents salivary contamination as well as includes a one-way valve to avoid the inhalation and condensation of ambient air through the condenser. What may be significant, especially in the context of asthmatic patients, is that the one-way valve prevents the inhalation of cold air by the patient through the condenser and then prevents unintentional cold air challenge [9]. Cooling temperature differs, ranging from zero to below −20 °C depending on EBC collection devices used [13,14,15]. The optimal temperature of collection remains unclear for many biomarkers. It has been found that lower temperature increases EBC collection rate, however, using the coldest sampling method decreased the ratio, probably due to the formation of a frozen layer on the sample surface, which impairs heat transfer [16].

In addition to the aspect of variable cooling temperature, a number of other methodological options that may affect the volume and composition of an EBC sample have been described. For example, it was noted that the EBC volume depends on the total surface of the condenser and the larger the condensing surface the higher the volume of EBC and the number of biomarkers detected [17]. Another observation was that the type of coating material may have a significant influence on the levels of different biomarkers found in EBC [18]. In order to minimize the reactivity of biomarkers with the collection devices, they contain an inert material on the condensing surface, e.g., Teflon, polypropylene, glass, silicone, or aluminum. The next important issue is the correlation between the volume of EBC and the total volume of exhaled air [19,20]. For standardization of EBC sampling, it would be advantageous to perform collection for a time over which a pre-defined volume of air is exhaled rather than setting a fixed collection time. Keeping the same, a pre-defined volume of the expired air would minimize the inter-individual variability of EBC volume.

Here we present the more detailed characteristics of collectors that have been most often used by researchers. EcoScreen is among the most frequently described condensers in the literature. It is a large collection device with an electrical cooling system that allows to maintain the temperature of the condenser. An optional spirometer is one of the additional features of the device. Although EcoScreen1 has been widely used in study protocols, it is no longer manufactured. A drawback to this collector was mainly the lack of manual control of condensing temperature. Moreover, the maintenance of the device was time-consuming because of the cleaning requirements between consequent trials. Its successor, EcoScreen2 is a modified version that collects larger sample volumes and higher concentrations of biomarkers than EcoScreen1 [21]. EcoScreen2 contains separate collection chambers allowing fractionated collection of EBC from different areas of the bronchi into disposable polyethylene bags. It includes an adjustable thermoelectrically cooled condenser, and the collection temperature may be as low as −20 °C.

The RTube breath condensate collection system differs significantly from the device described above. RTube consists of a disposable polypropylene tube acting as the condenser and collector that separates saliva from the exhaled breath and a one-way valve made of silicone rubber [22]. During the procedure of EBC sampling, the collector is placed into a pre-cooled aluminum sleeve. Typically, the sleeve is cooled to −20 °C and with the start of the procedure, the temperature gradually increases due to ambient temperature as well as that of the exhaled vapors. The construction of this device does not allow for maintaining the collection at a constant low temperature and therefore limits the time of effective collection. After sample collection has been completed, the mouthpiece should be disconnected from the device and the tube capped on both ends with rubber caps. The great advantage of this handheld, single-use device is that it can be used by unsupervised subjects.

The TURBO-DECCS is also a portable device that consists of a TurboUnit and a disposable single-use DECCS collection system [23]. The condenser includes a mouthpiece, one-way valve with saliva trap, a tube, and a collection cell inserted in a Peltier-type electrical cooling system. [21] The temperature of sampling is adjustable from 0 to −10 °C. It can be used both for conscious, non-ventilated patients as well as for those receiving invasive or non-invasive ventilation. According to the manufacturer, 1.5–3.0 mL of EBC samples can be obtained through collection. The manufacturer also offers a Pilot Study system which is very similar to RTube and requires a cooled aluminum sleeve.

The Anacon collection system is a device with a thermoelectric pump that has been used by many research groups [24,25]. The condensation temperature is within the range from −15 °C to 5 °C and it can be used in mechanically ventilated patients [24].

Summarizing the EBC collection devices’ properties: it is not easy to choose only one best commercially available system as each of them has its own characteristics (as described in the above section) and the decision should be made based on the study design.

## 4. Exhaled Breath Condensate—Sampling Procedure

EBC may be performed in actively participating patients of all ages breathing on their own, or in mechanically ventilated patients by placing a collection device in-line with the expiratory circuit of the ventilator [24]. EBC sampling does not have any influence on lung function or mediator levels, and it is believed that the collection may be successfully repeated many times with brief intervals [26,27]. Adverse effects have not been described even in children and adults with severe lung disease [26,27]. The procedure is performed in children as young as 3–4 years with the same technique that is used in adults. EBC sampling is manageable without the need for any specific medication, no external fluid is required to be added to the airways, comparing to BAL [28]. EBC may be successfully collected from newborn babies through a face mask [29]. Since the pattern of breathing is normal, EBC collection does not provoke bronchospasm in asthmatic patients and is even safer than forced vital capacity (FVC) measurements [9]. Although it has been reported that some individuals tend to hyperventilate especially at the beginning of EBC sampling, no adverse effects have been observed [9]. During the collection, patients are asked to stay in a sitting position and inhale and exhale through a special mouthpiece with a separated inlet (as an inhalation port) and outlet (toward the condensing surface) [9,13]. Various solutions are implemented to prevent salivary contamination of a sample, such as separating the mouthpiece from the condensing surface by the length of the tubing, placing the device at a higher level than the mouth, or using a mouthpiece with a salivary trap. Additionally, periodic swallowing is advised during the collection to reduce the risk of salivary contamination [13]. The nose-clip is applied to prevent inhaling of ambient air through the nose and the possibility that mediators produced locally in the nasal mucosa enter the inhaled air. It also reduces air leakage from the lower airways through the nose and mixing of nasal and bronchial air which would affect the composition of the EBC [9,13]. However, possible advantages of nasal inhalation, including a greater volume of condensate, and no influence on the levels of mediators were shown in healthy volunteers [19]. The exhaled air passing through a Teflon or polypropylene tube inside a cooling container is converted to exhaled breath condensate in the form of droplets or solid-phase material. Slow breathing cycles with quiet tidal breathing that does not affect lung function are recommended. There is no need for forced respiration because the larger the proportion of dead space ventilation, the higher EBC dilution and the greater the influence of ambient air [30]. Diurnal variation of the biomarkers has been described for H_2_O_2_ level in EBC of healthy subjects and patients with chronic obstructive pulmonary disease (COPD) [31,32].

There have been some attempts in rapid and prolonged collection time, ranging from 3 to 60 min [9,33,34]. The time of 10 min may result in 1–2 mL of condensate from adult subjects and most children above 4 years of age. It was indicated that subjects usually tolerate this period of collection without fatigue [9]. When the procedure is performed in children, the main difficulty is their loss of interest, so sample collection time should be minimized. 

The temperature and humidity of ambient air can directly affect the composition of EBC, which is why environmental conditions (temperature and humidity) should be measured [13]. Furthermore, water loss depends on humidity and ambient temperature. So far, none of the condensers used in study protocols collects all exhaled water vapor. Pollutants in ambient air may lead to inflammatory and biochemical changes in the airway that are reflected by alterations in the composition of the EBC [13]. Furthermore, atmospheric compounds may directly penetrate the obtained sample and interact with the molecules trapped in EBC, especially when the sample is left in an open tube.

Special attention should be paid to the influence of the upper airways on sample composition, especially in patients with current upper airway disease. The ongoing inflammation in the airways alters EBC composition [19]. Therefore, the exclusion criteria for participation in each study should be precisely defined in the study protocol. The research protocol should also include activities, such as exercise, food intake or smoking before EBC collection, as these factors may also be responsible for alterations in EBC concentrations of compounds [9,13]. Cigarette smoking is considered to be an important confounding factor although the effects of different cigarette brands, cigar or pipe smoking, have not been explored. In previous investigations, the increase of condensate levels of hydrogen peroxide, 8-isoprostane, prostaglandin E2, and leukotriene B4, and reduced levels of nitric oxide metabolites and a decrease of EBC pH were reported [35]. For this reason, participants should be advised to refrain from smoking at least 3 h before collection and smoking habits should be documented carefully in the study protocol [9]. Exercise withdrawal is also recommended at least one hour prior to the collection of EBC [36]. Coughing (either spontaneous or induced) and crying during collection may influence the composition of EBC, but the underlying mechanism has not been fully explained [37]. Further studies are needed to assess the effect of breath-holding on mediator levels in EBC.

**Figure 1:**
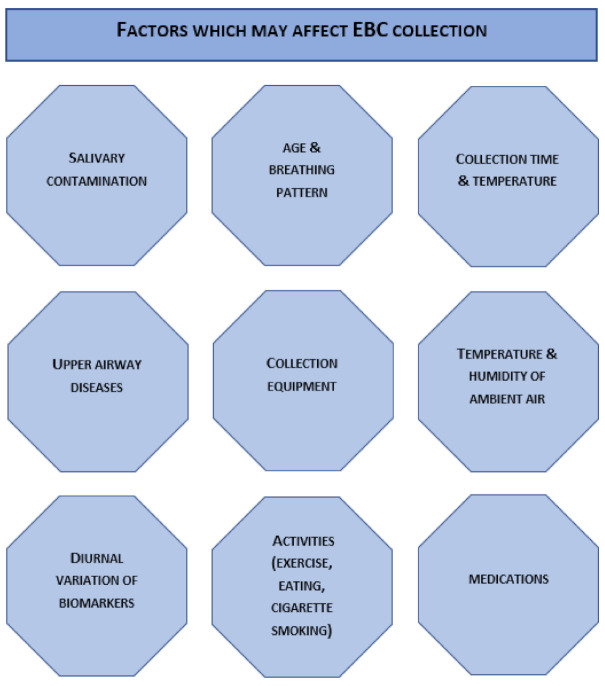
EBC collection influencing factors.

Some differences depending on the age of subjects have been detected in various EBC biomarkers although the published data are conflicting and further investigations are required [9]. No correlation has been observed between body weight and height and EBC volume or concentration of certain biomarkers (hydrogen peroxide) in adults [20]. The potential effects of race, gender, and body position on the volume or biomarker concentrations in EBC have not been elucidated [9]. Several studies have investigated the effect of medication on EBC composition however, a number of these studies lack a long-term controlled design and therefore further studies are needed [13]. Drug therapy in patients enrolled in the study should be carefully described and its effect on mediator levels should be considered. The utility of EBC for the assessment effects of therapeutic strategies will depend on the results of longitudinal studies. The results of the studies described in this section are not easy to compare because of the large number of methodological variants of sampling and potential confounding factors (Figure 1), which were not always explained in detail in the research methodology.

## 5. Analysis of Exhaled Breath Condensate

EBC contains a wide range of mediators of inflammation, oxidative stress, and nitrosative stress whose analysis may improve the diagnosis and management of patients with certain pulmonary diseases [10,11,12,21]. EBC consists of many biomarkers diluted in a water matrix, with different chemical stabilities [9]. The conditions and duration of storage may influence the concentrations of biomarkers [38,39]. For many biomarkers, assays are commonly at or near their detection limits, leading to higher variability [9,38,39]. A potential option to overcome this problem is the concentration of samples [9,13]. Exceptions in which levels are fully in the range of the available assays, include total protein level, nitrate, pH and ammonia [9]. Unstable volatiles in EBC during and after the procedure can be evaporated and released from the sample. H202 stability and partial pressure of CO2 in samples tend to decrease in time, which leads to changing sample composition [38]. Therefore, taking measurements of those two compounds is recommended in real time or immediately after collection [39].

The dominant component of EBC is condensed vapor (99.99%), containing volatile compounds [21]. The remaining liquid, in the form of droplets, contains non-volatile compounds of the airway lining fluid (ALF), such as different mediators but also microbes and metals [13]. The mechanism of recruiting these compounds is not clear yet, however, the two most widely accepted hypotheses explain it either by turbulent flow which causes small amounts of ALF to release from airway lumen surface [13], or by the recruitment of atelectatic areas of airways [26]. EBC dilution indicator should be established to determine actual levels of biomarkers in airways [21]. So far, no standard dilution factor has been found and this material cannot be considered as a standardized biological specimen [13]. Different collection systems and procedures will generate differently diluted condensates, despite similar concentrations in exhaled breath and, consequently, variable methods of concentrating or different extraction compounds are applied for biochemical analysis [13]. Lyophilization of EBC may be a potential method to concentrate the sample [40], however, the selected method should be adequate for the type of investigated biomarkers. For example, a recent study conducted in ten adult healthy participants investigated the significance of different physical and chemical modifications to improve the quality and quantity of the DNA extracted from EBC [41]. The results suggested that some methods, such as sodium acetate precipitation, using oligo (dT) primer, incubation at high temperature, or sodium dodecyl sulfate (SDS) treatment may be a new approach for the methods of measuring DNA concentration in EBC samples [41]. The method of EBC sampling itself entails the risk of contaminating the specimen from the lower airways with mediators or proteins from the upper airways, saliva and oral cavity [19]. A few solutions have been found, such as a saliva trap, 4.5% sodium bicarbonate mouthwash, or periodic swallowing by the subject during sample collection [26,42]. To exclude salivary contamination, measurement of salivary amylase has been proposed, since it has an impact on pH [43]. After proper precautions, amylase is found at a 10,000-fold lower concentration than in saliva [44].

Samples for the assessment of non-volatile compounds should be stored immediately after collection at a temperature of −80 °C until analysis [45]. It has been recommended to analyze the material as soon as possible after collection [13]. It is advisable to test the stability of mediators at storage temperature. Assays should be performed within the time during which the biomarker is known to be stable [9,13]. The time of storage for most of the compounds of EBC has not been determined yet, studies have shown no correlation between cytokine levels and time in a 1-year prospective study, though for leukotrienes significant degradation has been found after 3 months of storage [46].

Among the various components tested in the EBC, the possibility of detecting individual metals seems to be notably interesting because their concentration in EBC may reflect their translocation from the lung into the systemic circulation [47] and, also, cationic metals are involved in the control of airway smooth muscle tone [48]. In the studies conducted in healthy adults, the highest concentrations were found for zinc and iron in EBC. The levels of metals such as aluminum, cadmium, chromium, and tungsten in the majority of the investigated samples were below the limit of detection [47]. Metals in EBC may be quantified using graphite furnace atomic absorption spectrometry (GFAAS) and inductively coupled plasma mass spectrometry (ICP-MS). ICP-MS, compared to GFAAS, can measure more elements at a single test (over 75) and demonstrates a better linearity of range and in-run precision. However, the disadvantages of the method are its higher cost and larger volume of sample requirement [47].

## 6. Biomarkers in Exhaled Breath Condensate of Asthmatic Patients

Numerous mediators of airway inflammation, oxidative stress and nitrosative stress such as pH, hydrogen peroxide, isoprostanes, cytokines, leukotrienes, prostanoids, nitrogen oxides, and peptides have been studied in EBC samples of asthmatic patients [21,35,49].

### 6.1. Acidity

The role of EBC pH measurement as a potential prognostic factor in asthma remains uncertain. Many studies underlined the fact that while lower pH was observed among asthmatic patients, no differences in pH have been found in some of them [50,51]. Low EBC pH was obtained especially in patients with exacerbations, which suggests that it can be used for the detection of severe or uncontrolled disease [52]. The gradation of decrease in pH due to disease control level was also observed [53]. Wood et al. showed the influence of Mycoplasma pneumonia infection in asthmatic children on EBC pH [54], but their follow-up study did not confirm the primary thesis [55]. Pregnancy did not alter the acidity of airways in asthmatic women [56]. Active smoking, which is connected with worse asthma management, lowered pH in samples [57,58]. In a study conducted in asthmatic children, it has been demonstrated that asymptomatic gastroesophageal reflux did not affect pH of EBC [59] and the same authors also concluded that based on their results, EBC pH is not an informative tool in the assessment of childhood asthma.

### 6.2. Metals

As previously mentioned, it is possible to determine the concentration of some metals in EBC. Magnesium as a cation inhibits smooth muscle wall contraction [60] and for this reason it was proposed in asthma exacerbation management, in certain cases [61]. There is evidence of lower magnesium levels in EBC of asthmatic children [62]. As a potential biomarker of bronchoconstriction, its concentration in EBC was also investigated in patients suffering from bronchiolitis. Demirkan et al. conducted magnesium analysis in EBC obtained from infants with bronchiolitis and compared it to healthy children. The authors concluded that there were no differences in magnesium levels between bronchiolitis patients and healthy controls and, contrary to their hypothesis, the association between magnesium level in EBC and disease severity was not revealed [48].

### 6.3. Oxidative Stress

The concentration of hydrogen peroxide as a potential marker of oxidative stress was widely investigated in EBC [40]. Hydrogen peroxide is released by inflammatory cells as an outcome of respiratory burst [40]. H_2_O_2_ levels were elevated in asthmatic patients, regardless of age [63,64], and even after pharmacological treatment patients with asthma presented higher levels of this compound in EBC [63]. H_2_O_2_ concentrations in EBC increased in steroid-naive asthma and were influenced by the smoking habit [65] and disease treatment [66,67]. An in-house assessment of H_2_O_2_ levels in EBC could be a promising evaluation method of asthma management and there was a successful attempt of using smartphones for analytic methods [68].

Another biomarker of oxidative stress that has been investigated in EBC is 8-isoprostane, a prostaglandin-like molecule, produced in arachidonic acid peroxidation [69]. Its higher concentration has been previously found in BAL of asthmatic patients [11]. This compound has been also investigated in EBC and most of research papers pertaining to asthmatic children have indicated higher levels of 8-isoprostane compared to healthy controls [70,71,72], while no statistical significance has been found in some of them [73]. Its levels in EBC substantially decreased after oral prednisone treatment [70]; although even after obtaining asthma symptoms, the control 8-isoprostane concentration remained higher than in healthy individuals. This compound has been a subject of systematic reviews in both pediatric and adult populations, but due to variability of conclusions and inefficient amount of research, its clinical status remains uncertain [74].

### 6.4. Cytokines

Cytokines have been the most researched molecules in terms of the usefulness of EBC in asthma diagnosis [49]. Interleukin 2 (IL-2) is a cytokine responsible for the proliferation of all T cells and it is involved in both pro-inflammatory and regulatory pathways [12,75]. In EBC samples it was elevated in asthmatic patients comparing to the healthy control group, although there was no correlation between disease severity and IL-2 levels [76]. Among the non-atopic asthmatic group, a reverse correlation between asthma control test score, forced expiratory volume in 1 second (FEV1), and IL-2 has been detected [76]. Th-2-related cytokines—interleukin 4 (IL-4), interleukin 5 (IL-5), interleukin 9 (IL-9), and interleukin 13 (IL-13)—play an important role in the pathophysiology of inflammation [77,78]. IL-4 levels in EBC were elevated in asthmatic children, compared to the healthy group [79]. Its EBC concentration was negatively correlated with the dosage of inhaled corticosteroids (ICS) [80]. IL-5 concentration was also higher in asthmatic patients than in healthy individuals [79], as well as in atopic than non-atopic patients [81]. In a prospective study by van Vliet’s et al., a group of asthmatic children were observed for exacerbations over a one-year timeline, and no inflammatory marker levels in EBC, including IL-5, interleukin 6 (IL-6), interleukin 8 (IL-8), and IL-13 were useful as exacerbation predictors [82]. Cytokines were not always available for detection in the samples of children’s exhaled breath condensates [83]. IL-6 is a cytokine produced by the cells of the immune system, but also by primary pulmonary endothelial cells due to various airway stimuli; its higher levels were detected in EBC of asthmatic adults [84]. In one study, the EBC levels of IL-6 were elevated also in asthmatic children comparing to healthy ones [85]. No significant differences in IL-6 EBC samples were found between asthmatic adults and COPD patients [86]. IL-8 is a chemotactic cytokine, involved in both acute and chronic inflammation processes [87]. COPD patients during exacerbation tend to present higher IL-8 levels than asthmatic patients, both in serum and EBC [88].

### 6.5. Leukotrienes

Cysteinyl-leukotrienes (Cys-LTs; LTC4, LTD4, LTE4) are inflammatory mediators produced from arachidonic acid by eosinophils, mast cells, basophils and macrophages; they are among the strongest bronchoconstrictors [89]. Cys-Lts in children’s EBC were elevated in the asthmatic group comparing to the non-asthmatic group, as well as in persistent compared to intermittent asthma [90]. On the contrary, in a study by Keskins et al. on asthmatic children, no correlation has been found in Cys-LT levels with regard to asthma severity, control, and the Childhood Asthma Test score [91]. However, in a study comparing oral prednisone and inhaled single high-dose fluticasone propionate (4000 ug) treatment, a significant decrease of cysteinyl leukotrienes was found in exhaled breath condensates in both groups four hours after receiving medications [92]. Leukotriene B4 (LTB4) is another leukotriene synthesized from arachidonic acid; binding with its receptor on T cells, it induces cytokine excretion [93]. Trischler et al. fractioned EBC with the use of Eco-Screen 2 from the large and small airways; samples from small airways in asthmatic children had significantly higher LTB4 levels than in healthy ones [94]. Also, in atopic patients, LTB4 concentration was higher than in non-atopic ones in both types of collected EBC.

### 6.6. Enzymes

Matrix metallopeptidase 9 (MMP-9) is a protease involved in the degradation of the extracellular matrix [95] and tissue restoration [96]. It is involved in airway remodeling in asthma and COPD [97]. A higher concentration of MMP-9 in EBC has been found in children with allergic asthma and it has correlated with the total immunoglobulin E serum level of these patients [98]. However, another study in children did not confirm its importance as a potential distinguisher between atopic and non-atopic asthma [81]. In another study, increasing the intake of inhaled corticosteroids in asthmatic children did not result in a significant reduction of metalloproteinase, although the study group consisted of only four people [99].

### 6.7. Nitric Oxide Products

Nitric oxide products in EBC are a promising marker [100]. EBC levels of asymmetric dimethylarginine (ADMA), an inhibitor of nitric oxide synthase, are elevated in asthmatic patients, however, its concentration does not correlate with lung function parameters and its serum concentration [45,69]. Total nitric oxide products, i.e., nitrate and nitrite, are more stable endpoints of NO metabolism which can be found in EBC. Patients with atopic asthma and poly-aero-sensitization showed increased NOx concentrations [101].

### 6.8. Hormones

Leptin, a hormone connected with body weight regulation, exerts the effect on T-lymphocyte response—it promotes Th1 phenotype while suppressing Th2 which may be one of the elements of atopic asthma development [102]. Serum leptin levels were higher among asthmatic children, especially in boys. Unfortunately, in two studies with the use of EBC samples, there were conflicting conclusions; one of them confirmed a higher level of leptin among asthmatic and obese patients [103], while another did not manage to show a sufficient amount of this hormone in condensates [104].

### 6.9. Proteins

Periostin is a matrix protein, expressed in fibroblast and epithelial cells, that seems to be an adequate biomarker in asthma because it is involved in the Th2 inflammatory response. Its increased production was observed in patients with allergic rhinitis [105]. There are reports of its higher level in the serum of children with severe asthma comparing to the control group [106,107] and in asthmatic patients with dynamic hyperinflation, which decreased inspiratory capacity during tachypnoea [108]. During omalizumab therapy, periostin concentration decreased more than in treatment with conventional corticosteroids [109]. The serum level of this molecule can be falsely elevated in children because it is released by osteoblasts during growth. In a study of adult asthmatic patients, EBC levels of periostin were more useful to determine the severity of upper respiratory tract infections, while serum levels were more accurate for the assessment of asthma activity [110]. Its EBC levels were higher among asthmatic patients with comorbid chronic rhinosinusitis, especially within patients with positive bacteria cultures from nasopharyngeal swabs, but were not correlated with asthma control, asthma severity or the intensity of ICS treatment. A study by Nejman-Gryz et al. concerning children with mild asthma confirmed the limited usefulness of EBC periostin in terms of the detection and management/control of asthma [111].

### 6.10. Micro-RNAs

Non-coding RNAs are involved in many biological pathways. They are divided into housekeeping and regulatory RNAs. From the regulatory group which affects the immunological responses, micro-RNAs (miRNAs) are the main interest of studies in terms of respiratory diseases [112]. In experimental studies, numerous miRNAs were connected to allergic asthma. A study by Pinkerton et al. was the first to prove a possibility to isolate a sufficient amount of miRNA in EBC and confirmed the downregulation of Th2-response mediators, such as miR-21, miR-155, miR-133a, and miR-1248 in airways [113]. A recent study by Mendes et al. showed a possibility to assess asthma endotypes in EBC material of pediatric patients through miRNAs. Certain miRNAs were associated with symptomatic asthma and correlated with bronchodilator response [114].

The biomarkers detected in exhaled breath condensate are summarized in Table 2.

## 7. Microbiota in Exhaled Breath Condensate

Microorganisms have a significant impact on both the prevalence and exacerbations of asthma. Recent studies have shown that the lower airways of healthy individuals are inhabited mostly by five major bacterial phyla: Proteobacteria, Firmicutes, Actinobacteria, Fusobacterium, and Bacteroidetes [115]. Both quality and quantity differences are observed in the bacterial microbiome of asthmatic patients. People suffering from this disease had a higher abundance of species belonging to the Proteobacteria phylum, such as the Nitrosomonadaceae, Oxalobacteraceae, Pseudomonaceae, and Pastereullaceae families, and a lower abundance of the Bacteroidetes and Firmicutes phyla [115]. There are several studies to date on bacterial and fungal assessment with the usage of exhaled breath condensates.

A study by Glendinning et al. compared EBC and protected specimen brushings (PSB) in an animal model. EBC samples had significantly less DNA material, which is the main limitation of the method [116]. The advantage of exhaled breath condensates is non-invasive sample collection. Moreover, the differences in bacterial species of those two sampling methods can be explained by the fact that PSB material is collected from a limited small region of the larger airways, such as trachea and bronchi, when EBC seems to include microbiota from the smaller airways as well. Given the fact that EBC bacterial load is too low for standard cultures, the taxonomic marking must be conducted with DNA PCR amplification with certain 16s rRNA primers, to further determine certain types of bacteria.

A study by May et al. compared BAL with EBC samples in the detection of bacterial DNA in patients with Ventilator-Associated Pneumonia. Both sample types had almost identical concordance (>95%) in pathogen DNA detection [117].

Researchers tested the feasibility of EBC for both bacterial and viral detection in COPD patients. Although DNA was obtained in most of the samples, the detection outcome differed from sputum samples [118].

In a study by Carpagnano et al., colonization by Aspergillus niger, Aspergillus ochraceus and Penicillum spp. in the airways of lung cancer patients was detected with the use of EBC [119]. In asthmatic patients, the airways were colonized substantially more than within the cancer group (70% of patients), mostly by Cladosporium (94% of the asthmatic group), Alternaria (21%) and Penicillium (24%) species, whereas no colonization was found in the control group [120].

It has been noticed that people with uncontrolled and severe asthma had an increase of fungi colonization in airways [120]. Exhaled breath condensates had identical sensibility compared to both bronchial brushing [119] and induced sputum [120] for fungi detection. In contrast to the previous studies, noticeable percentage of airways fungi has been detected also among healthy volunteers in Italy, with dominance of Aspergillus sydowii and Cladosporium spp., although due to possible ambient air pollution and being a single report, the hypothesis of fungal airway presence among healthy ones remains uncertain [121]. The fact that asthmatic patients’ airways compared to lung cancer patients were more colonized by fungi, highlights the need for future studies of microbiome as a potential factor of uncontrolled disease.

The studies showed less utility of EBC in viral detection Condensates have almost non-existing sensitivity in both multiviral detection panels and influenza tests [122,123]. A promising data from COVID-19 pandemic indicate possible usefulness of breath condensates in disease laboratory confirmation. Ryan et al. collected EBC samples from patients with clinical diagnosis of COVID-19 with negative nasopharyngeal swab SARS-CoV-2 outcome, in which EBC results had high specificity for this virus detection [124].

## 8. Conclusions

Exhaled breath condensate (EBC) is an easy to collect, non-invasive, and affordable biological material which may allow for a better understanding of the pathology of asthma disease. The technique of exhaled breath condensate collection does not disturb the ongoing respiratory inflammation in contrast to such procedures as sputum induction or bronchoalveolar lavage. EBC offers the possibility of safe and repeated measurements in all groups of ages for long-term follow-up. The possibility of sampling from preschool children allows for developing a new objective method for the detection of the disease among the population with recurrent wheezes. It would also help with early prediction of asthma exacerbations. Inflammation and oxidative stress mediators, microbiome, and proteomic and metabolomic composition in asthma, can be studied [125,126]. The differences in the methodology of EBC collection and analysis may limit the possibility of comparing published data, but some recommendations were published to facilitate standardization. In 2005, the American Thoracic Society (ATS) and the European Respiratory Society (ERS) created a task force on EBC to determine guidelines in exhaled breath condensate collection and analysis [9]. In 2010, the ERS published a monograph on exhaled biomarkers [127]. Recommendations on the EBC sample collection procedure and technical standards of EBC analysis have been published in 2017 by the ATS/ERS and serve as a guide for future studies [13]. The relevance of future EBC publications depends not only on the scientific description of biomarkers detected in particular groups of individuals, but also on the meticulous description of the protocol used to obtain, preserve and analyze EBC. The analysis of exhaled air also requires more attention to be paid to the human microbiome and its confounding effect on breath biomarkers. Therefore, there is a need for large surveys in well described groups of asthmatic patients to finally determine the place of EBC in the diagnosis of asthma. Due to the progress in laboratory techniques of the detection and analysis of new markers from condensate, it seems to be a promising material for a better understanding of asthma pathology and management.

## Figures and Tables

**Table 1:** Types of condensing equipment.

Name of Device	Producer	Cooling System	Specific Features
EcoScreen1	Cardinal Health, Hoechber, Germany	Electrical cooling System	Not currently manufactured
EcoScreen2	FILT Lungen-& Thorax Diagnostik GmbH, Germany	Electrical cooling System	Fractionated collection possible
RTube	Respiratory Research, USA	Pre-cooled sleeve sensitive to higher ambient temperature	May be used at home by unsupervised subjects
TurboDECCS	Medivac, Italy	Electrical cooling System	Fractionated collection possible
ANACON	Biostec, Valencia, Spain	Electrical cooling System	May be used in mechanically ventilated patients

**Table 2:** Biomarkers detected in EBC.

Fields of Interests	Biomarkers
Acidity	pH
Metals	Magnesium
Oxidative stress	H_2_O_2_, 8-isoprostane
Cytokines	IL-2, IL-4, IL-5, IL-6, IL-8, IL-9, IL-13
Leukotrienes	LTB4, LTC4, LTD4, LTE4
Enzymes	MMP-9
Nitric oxide products	ADMA, NOx
Hormones	Leptin
Proteins	Periostin
Genetics	miRNAs

## Data Availability

Not applicable.
